# Cremation in France

**Published:** 1886-05

**Authors:** 


**Cremation in France.**—The French parliament at a late session, be a vote of 321 to 174, adopted in the form of an amendment to the law relative to funerals, the principle of cremation, by according to every minor or adult, the right to determine by will, the disposition of his mortal remains, as between interment and incincration.

The "Journal d' Hygiene is commenting upon this forward step, reminds its readers that for more that ten years the Journal has been a constant advocate of incineration as a needed sanitary reform.

